# Clinical efficacy of an ultrasound-guided bilateral rectus sheath block for umbilical hernia repair in calves: A prospective randomized trial

**DOI:** 10.3389/fpain.2023.1051504

**Published:** 2023-02-13

**Authors:** Fabiana Micieli, Jacopo Guccione, Giovanni Della Valle, Maria Chiara Alterisio, Paolo Ciaramella, Giancarlo Vesce, Ludovica Chiavaccini

**Affiliations:** ^1^ Department of Veterinary Medicine and Animal Productions, University of Napoli Federico II, Napoli, Italy; ^2^ Department of Comparative, Diagnostic, and Population Medicine, College of Veterinary Medicine, University of Florida, Gainesville, FL, United States

**Keywords:** calves, herniorrhaphy, locoregional anesthesia, rectus sheath block, umbilical hernia repair

## Abstract

**Introduction:**

Surgical umbilical hernia repair is a frequent procedure in newborn calves, requiring mandatory pain management. This study aimed to develop an ultrasound-guided rectus sheath block (RSB) and to evaluate its clinical efficacy in calves undergoing umbilical herniorrhaphy under general field anesthesia.

**Methods:**

Gross and ultrasound anatomy of the ventral abdomen and the diffusion of a new methylene blue solution after injection within the rectus sheath were described in seven fresh calf cadavers. Then, fourteen calves undergoing elective herniorrhaphy were randomly assigned to receive either bilateral ultrasound-guided RSB with 0.3 mL/kg of bupivacaine 0.25% and 0.15 µg/kg of dexmedetomidine or 0.3 mL/kg of 0.9% NaCl (control). Intraoperative data included cardiopulmonary variables and anesthetic requirements. Postoperative data included pain scores, sedation scores and peri-incisional mechanical threshold assessed by force algometry at specific time points after anesthetic recovery. Treatments were compared using Wilcoxon rank-sum, Student's *t*-test, and Cox proportional hazard model as appropriate. Mixed effect linear models on rank, with random effect calf; fixed effects time, treatment, and their interaction were used to compare pain scores and mechanical thresholds over time. Significance was set at *p* = 0.05.

**Results and Discussion:**

Calves receiving RSB recorded lower pain scores between 45 – 120 minutes (*p* < 0.05) and at 240 min after recovery (*p* = 0.02). And they recorded higher mechanical thresholds between 45 and 120 min after surgery (*p* < 0.05). Ultrasound-guided RSB provided effective perioperative analgesia in calves undergoing herniorrhaphy under field conditions.

## 1. Introduction

In recent years, there has been a growing awareness of the importance of identifying and relieving stress and pain in farm animals. In addition to any ethical consideration, it is now well accepted that adequate control of perioperative pain is of high importance, as the release of nociceptive neuroendocrine transmitters can influence surgical outcomes (1, 2). Despite this rising awareness, the systemic use of analgesia for surgical procedures in livestock is erratic (3) due to the perception that pain management may be associated with increased costs more than gains due to improved productivity (4, 5). In addition, there is a widespread misconception that younger animals are less sensitive to pain than adults of the same species. However, studies show that age has no influence on acute pain development in animals undergoing tail docking and castration (6) or cautery disbudding (2, 7, 8).

Umbilical diseases requiring surgical intervention are frequent in calves (9, 10). According to a recent survey, nonsteroidal anti-inflammatory drugs (NSAIDs) remain the mainstream analgesics administered to cattle in the perioperative period (3, 11). They have been proven beneficial in several surgical procedures such as castration and disbudding (5, 12), but they may not be effective in the immediate perioperative period (13). Regrettably, only a few NSAIDs are approved by the U.S. Food and Drug Administration and the European Union for restricted use in bovine (14, 15). Constant rate infusions (CRIs) of lidocaine alone (16) or in association with morphine and ketamine (17) have been proven effective in providing analgesia during umbilical surgery under general anesthesia in calves. However, the technical support required for CRI administration limits its use in hospital settings. In field conditions, locoregional anesthesia is the most common technique to provide analgesia in livestock (5). Locoregional techniques are easy to perform and economically sustainable and have the advantage of peripherally blocking the transmission of pain signals without systemic effects. Locoregional anesthesia of the abdominal wall around the umbilical incision could effectively provide perioperative analgesia during umbilical surgery in calves.

Descriptions of bovine abdominal wall innervation are scarce and mostly focused on the flank. However, it has been described that in cattle the sensation around the umbilicus is supplied by the ventromedial branches of the 10th–12th thoracic spinal nerves (T10–T12) that run caudoventrally to the last ribs between the two intercostal muscles, to emerge between the internal oblique and the underlying transverse abdominis muscle. The nerves then proceed in a caudoventral direction and pierce the rectus abdominis muscle and the aponeurosis of the internal and external abdominal oblique muscles to innervate the skin anterior to the umbilicus (18, 19). The rectus muscle originates from the outer surface of the fourth to ninth costal cartilages and extends to the pubic region (19, 20). In cattle, unlike dogs, the aponeurosis of the external and internal oblique muscles forms the external lamina of the sheath of the rectus muscle, while only the aponeurosis of the transverse abdominis muscle forms the internal lamina of the sheath (20). A potential space exists between the internal border of the rectus muscle and its internal sheath, where a local anesthetic agent can be deposited to block the ventral rami of T10–T12 spinal nerves. This approach takes the name of rectus sheath block (RSB), and it was first suggested by Ferguson et al. (21) for providing perioperative analgesia in children undergoing herniorrhaphy. Bilateral RSB with 0.1 ml/kg 0.25% levobupivacaine provided sufficient perioperative analgesia in children undergoing umbilical hernia repair (22). In a more recent prospective, blind study, RSB appeared to improve postoperative analgesia and pain scoring with respect to the sole local anesthetic infiltration in a similar population (23). To our knowledge, the anatomy of the RSB has been described in thawed canine (24) and bovine (25) cadavers, but its clinical efficacy has never been tested in veterinary species.

The aims of this study were (1) to describe the RSB technique in fresh calves’ cadavers and (2) to evaluate the clinical efficacy of bilateral RSB in controlling perioperative pain in calves undergoing herniorrhaphy under general anesthesia in field conditions. We hypothesized that (1) the rectus sheath could be easily identified and targeted with high-frequency ultrasonography and an in-plane technique. We further hypothesized that (2) bilateral RSB with 0.25% bupivacaine hydrochloride (HCl) would improve postherniorrhaphy pain assessed with a validated composite pain scale and with pressure algometry with respect to a sham injection in calves.

## 2. Materials and methods

To determine an acoustic window for the ultrasound-guided needle placement in live animals, first, we evaluated the ultrasonographic anatomy of the ventral abdomen and the diffusion of a new methylene blue solution after RSB in seven fresh calf cadavers, dead for reasons unrelated to the present study. After the anatomical study, we tested the efficacy of the RSB on 14 live calves of various breeds undergoing elective herniorrhaphy under anesthesia in field conditions.

### 2.1. Anatomical study

**Figure 1:**
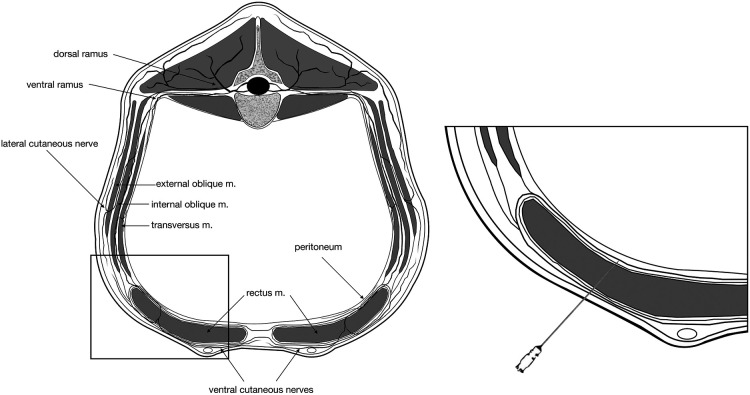
Schematic representation of the landmarks for injection in the internal rectus sheath plane. For simplification, only one spinal nerve is represented. It should be noticed that the sensation around the umbilicus is supplied by the ventromedial branches of the tenth to twelfth thoracic spinal nerves (T10–T12) that run caudoventrally to the last rib and then proceed in a caudoventral direction and pierce the rectus abdominis muscle and the aponeurosis of the internal and external abdominal oblique muscles to innervate the skin anterior to the umbilicus.

**Figure 2:**
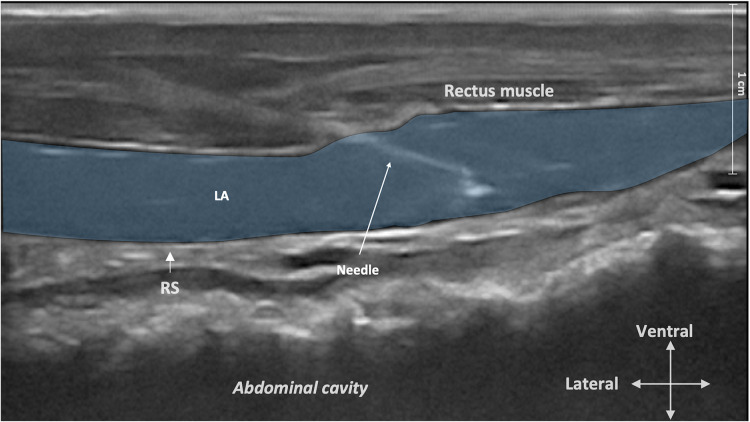
Ultrasound image of the rectus sheath block injection. The ultrasound transducer was placed in a transverse orientation to the midline before sliding it laterally to the *umbilicus* to identify the rectus abdominis muscle, the twin “tram” lines, and the peritoneum. The needle tip was then advanced in the mediodorsal direction through the rectus abdominis muscle until it reached the plane between the rectus abdominis muscle and its internal sheath. LA, local anesthetic; RS, rectus sheath.

The use of bovine cadavers for this part of the study did not require ethical review or approval as per the Ethical Animal Care and Use Committee of the University of Naples Federico II. A group of seven stillborn calves from two local dairy farms, delivered within 24 h and maintained in a temperate environment (but not refrigerated), were positioned in dorsal recumbency. The ventral aspect of the abdomen was clipped to 30–40 cm around the umbilicus and prepared with alcohol. To identify the anatomical landmarks and the correct injection site, a portable ultrasound device (MyLab DeltaVET—Ultrasound; ESAOTE S.p.A., Italy) equipped with a high frequency (8–13 MHz) linear-array transducer was used. To identify the rectus abdominis muscle, the transducer was initially placed in a transverse orientation to the midline to identify the *linea alba* before sliding it laterally to the umbilicus. The area was scanned in a medial-to-lateral direction until the following landmarks could be visualized: (1) the lateral aspect of the rectus abdominis muscle; (2) the twin “tram” lines representing the internal lamina of the sheath of the rectus abdominis muscle and the transversalis fascia; and (3) the peritoneum (24–26). Once the operator became confident with the ultrasound anatomy, a 22-gauge, 90-mm spinal needle (Spinale Quincke, PicIndolor; Artesana S.p.A., Italy) was introduced in a lateral-to-medial orientation and at a 30° angle with the in-plane technique. The needle was connected to a 50-ml Luer-lock syringe (50 ml syringe; Terumo Italia S.r.l., Italy) through a 25-cm extension tube (Discofix; BBraun Milano S.p.A., Italy) and primed with a 1 : 1 solution of methylene blue (Nuovo blu di metilene; Bio Optica Milano S.p.A., Italy) and 0.5% bupivacaine HCl (Bupivacaina Recordati; Recordati S.p.A., Italy). The needle tip was then advanced in a mediodorsal direction through the rectus abdominis muscle until it reached the plane between the rectus abdominis muscle and its internal sheath (Figures 1, 2). A test dose of 1–2 ml was administered, and the position of the needle was considered adequate if hydrodissection separated the rectus abdominis muscle from the internal rectus sheath as described by James et al. (24). If the position was correct, a volume of 0.3 ml/kg of dye solution was injected under ultrasound visualization (Figure 2). If the position was deemed incorrect, the needle was adjusted and another test dose was administered. Immediately after injection, the cadavers were dissected. The skin and the subcutaneous tissue were incised to expose the *linea alba* and reflected laterally. Subsequently, the rectus abdominis muscle was carefully isolated, preserving the internal rectus sheath and the peritoneum. During dissection, we were unable to expose the ventral branches of the spinal nerves in some of the cadavers; consequentially, only the craniocaudal distribution from the umbilicus and lateral spread of the dye solution from midline were measured (in cm) with a caliper (Stainless steel digital caliper; Neiko, ZJ, China) and recorded.

### 2.2. Clinical trial

The clinical trial was performed on 14 cow calves affected by an umbilical hernia and referred to the Didactic Clinical Mobile Service belonging to the Veterinary Teaching Hospital of the Department of Veterinary Medicine and Animal Production of Napoli (Italy) from May 2018 and June 2020. The study received institutional approval from the Ethical Animal Care and Use Committee of the University of Naples Federico II (PG/2018/0050013). All the procedures performed during the study abode by the common good clinical practices and were performed by expert clinicians. Finally, the owners were informed and in agreement with the purposes and methods assessed. They also gave written consent for the study.

#### 2.2.1. Study design and population

The study was a prospective, randomized, blinded, placebo-controlled clinical trial. The sample size was based on the results obtained by Lomax and Windsor (27) comparing mechanical thresholds in calves castrated with or without the application of a topical anesthetic agent using von Frey filaments. Assuming an alpha error of 0.05 and a power of 0.8, we calculated that at least five calves per group would be required. Fourteen calves of various breeds undergoing elective herniorrhaphy under general anesthesia in field conditions were enrolled at their premises.

On the day of surgery, all calves underwent a complete physical and ultrasonographic examination of the umbilical hernia. Calves were deemed systemically healthy based on preanesthetic physical examination, complete blood count, and serum biochemistry. Calves were classified as physical status I or II according to the American Society of Anesthesiologists (ASA). Exclusion criteria included owner refusal, ASA physical status ≥III, intractable behavior, known or suspected coagulation impairment, skin infection at the site of injection or risk of systemic infection, suspect of the liver, renal, or gastrointestinal disease, or any contraindication for the use of nonsteroidal anti-inflammatory drugs or local anesthetic agents. For each calf enrolled in the study, food was withdrawn for 12–18 h, while water was withdrawn for 8–12 h.

#### 2.2.2. Anesthetic management

Calves were randomly assigned to receive either RSB or a sham injection by means of a random number generator (www.randomizer.org). Calves in the RSB group received a rectus sheath injection with 0.3 ml/kg 0.25% bupivacaine HCl (Bupivacaina Recordati; Recordati S.p.A., Italy) containing dexmedetomidine HCl (1 μg/ml; Dexdomitor 0.5 mg/ml; Zoetis Inc., United States) as an adjuvant to prolong the effect of the local anesthetic, as previously described (28, 29). Calves in the control group received a rectus sheath injection with an equivalent volume of sterile saline (0.9% NaCl). All injections were administered by the same operator (FM).

A 14-gauge catheter (Introcan Safety; BBraun Milano S.p.A., Italy) was aseptically placed in one of the jugular veins. Calves were allowed to rest in a quiet room for 120 min prior to induction of anesthesia. All procedures were performed in a dedicated clean area outside the barn. The area was protected from direct sun and well-ventilated. After premedication with an intravenous (IV) injection of 0.02–0.05 mg/kg xylazine (Nerfasin 100 mg/ml; Ati S.r.l., Italy) and 0.02 mg/kg butorphanol (Alvegesic 10 mg/ml; Dechra Veterinary Products S.r.l., Italy), anesthesia was induced with 2.5 mg/kg IV ketamine (Lobotor 100 mg/ml; ACME S.r.l., Italy). All calves were positioned in dorsal recumbency, raised from the ground, and laterally content by straw bales, with legs secured far from the surgical field and with the head elevated and the tip of the nose down to avoid aspiration. Intraoperative monitoring included heart rate (HR) determined by auscultation with a stethoscope, respiratory rate (*f*_R_) calculated by direct observation of the thoracic excursions, arterial hemoglobin saturation of oxygen (SpO_2_) measured with a portable pulse oximeter (CMS-50D1 Fingertip Pulse Oximeter; AccuMed, TX, United States), and rectal temperature. Data were continuously monitored and recorded every 5 min throughout the procedure. At baseline, skin incision, and at the end of surgery, a venous blood sample was collected in a heparinized syringe and analyzed immediately using an automated bench-top blood-gas analyzer (iSTAT 1 Analyser; VetScan, United States) for monitoring ventilation and electrolyte status.

If the calf responded to surgical stimulation with gross movement, spontaneous blinking, nystagmus, or increased jaw tone, additional boluses of IV ketamine (0.5 mg/kg) and/or xylazine (0.01 mg/kg) were administered. At the end of the surgical procedure, 1.1 mg/kg of flunixin meglumine (Alivios; Fatro S.p.A., Italy) was administered IV, and calves were positioned in sternal recumbency, with the neck extended forward for recovery. The time elapsed between the end of the surgery and the animal being able to hold sternal position without support (time-to-sternal) and the time from sternal recumbency to stand (time-to-stand) were recorded.

#### 2.2.3. Surgical procedure

After induction and when the animal was deemed at a surgical plane of anesthesia, the ventral surface of the abdomen was clipped from the xiphoid to the pubis and aseptically prepared prior to performing the block. After allowing 15 min for the block to impart clinical effect, each calf underwent a “closed umbilical herniorrhaphy” as described. Briefly, through an elliptical skin incision and a blunt dissection of the subcutaneous plane, the wall defect was exposed. The hernial ring edge was bluntly dissected from surrounding tissues and completely exposed. The internal hernial sac was gently replaced into the abdomen, and a finger was placed under the hernial ring edge to lift the wall avoiding underlying viscera damage. A slight incision around the ring was made, letting intact the hernial sac. A simple interrupted suture (Vycril USP 2 Ethicon Vicryl; Alcyon, Italy) was placed and progressively closed, avoiding extensive suture tension and the insertion of underlying tissues. The body defect was closed, and the subcutaneous tissues and the skin were closed in routine manners.

#### 2.2.4. Pain assessment

The same investigator (JG), experienced in bovine behavior and welfare and blinded to the treatment, was responsible for clinical assessment, behavioral pain scoring, and mechanical threshold determination. Each calf was evaluated for pain at baseline and 30, 45, 60, 120, 240, and 360 min after standing.

##### 2.2.4.1. Pain scores

Pain severity was scored using the UNESP-Botucatu scale, a unidimensional composite pain scale previously validated for cattle undergoing orchiectomy (30). The UNESP-Botucatu scale is reported in detail in Supplementary Table S1. Briefly, five behaviors were monitored by an ordinal scale, ranging from 0 (normal) to 2 (totally abnormal), and applied to each parameter. Sedation was subjectively scored with a simple 0–3 sedation scale (0, fully alert or walking; 1, mildly sedated; 2, moderately sedated; 3, asleep) to be accounted for in the analysis.

##### 2.2.4.2. Mechanical threshold

Peri-incisional mechanical threshold was assessed using a digital force algometer (FPX-100 Algometer; Wagner Instruments, United States). The algometer was applied perpendicular to the skin surface at four sites (right-cranial, left-cranial, right-caudal, and left-caudal) 1 cm lateral to the surgical incision (or around the hernia, at baseline), and pressure was applied until the calf showed signs of discomfort (i.e., moving away, kicking, arching the back). At that point, the algometer was removed, and the force (measured in N/cm^2^) that elicited avoidance behavior was recorded. A maximum cutoff pressure of 500 N/cm^2^ was used. Mechanical threshold tests were performed after the behavioral assessment of pain.

### 2.3. Statistical analysis

All analyses were conducted in Stata/IC version 14.2 for Mac (StataCorp, College Station, TX, United States). Data were tested for normality of distribution using the Shapiro–Wilk normality test and graphically with the normality quantile plot and histogram and reported as mean ± SD and median [interquartile range (Q1, Q3)] if normally or non-normally distributed, respectively. Demographic data and physiologic and intraoperative parameters were compared using Student’s *t*-test or the Mann–Whitney *U* test as appropriate. The time from the end of the procedure to the time the calf could hold sternal position and to the time the calf was able to stand were modeled using Cox proportional hazard survival models. Since repeated intraoperative doses of xylazine and ketamine may have affected the recovery time, we controlled for them by including both variables in the final multivariable regression model. Baseline pain scores and cumulative mechanical thresholds between calves receiving RSB and controls were compared using the Mann–Whitney *U* test. Baseline cumulative mechanical thresholds of the left and right hemiabdomens were compared using the Wilcoxon signed-rank test. For nonparametric data, the median difference between calves in the two treatment groups was calculated along with 95% confidence intervals (CIs) by using the Hodges–Lehman method, as proposed by Newson (31) and Iman and Conover (32). Data were then rank-transformed for further analysis (33). The differences in pain scores and cumulative mechanical thresholds between treatment groups over time were evaluated using a mixed-effects linear regression model, with the calf as a random effect and time and treatment as fixed effects. An interaction term between time and treatment was also included. Significance was set at *p* ≤ 0.05.

## 3. Results

### 3.1. Anatomical study

The anatomical landmarks considered in the present study were recognized, and the ultrasound images during injections were subjectively considered excellent in all cadavers. Under ultrasound, the rectus abdominis muscle was visualized as a hypoechoic structure surrounded by a hyperechoic rime identified ventrally (or superficial on the ultrasound image) as the external lamina of the sheath and dorsally (deeper on the ultrasound image) as the internal lamina of the sheath of the rectus abdominis muscle. The transversalis fascia was visualized as a hyperechoic line located dorsally to the internal lamina of the sheath of the rectus abdominis muscle. The peritoneum was identified deep into the transversalis fascia as a hyperechoic line. The RSB plane for injection was identified between the rectus abdominis muscle and its internal sheath, ventral (or superficial on ultrasound image) to the hyperechoic twin “tram” lines representing the internal lamina of the sheath of the rectus abdominis muscle and the transversalis fascia (24, 26).

In all cadavers, the injectate was correctly located between the rectus abdominis muscle and its internal lamina of the sheath. There was no staining within the abdominal cavity in any of the cadavers. The mean ± SD values for the craniocaudal and the mediolateral spread of the dye solution were 13.15 ± 2.62 cm and 5.8 ± 0.86 cm, respectively.

### 3.2. Clinical trial

Breeds included 10 Holstein Friesian, one Italian Braun, and three crossbreeds. There were eight females and six males aged (mean ± SD) 94.86 ± 29.76 days. Neither the proportion of females vs. males (*p* = 0.60) nor the age (*p* = 0.76) differed between groups. Calves’ body weights (mean ± SD) for RSB and control groups were 135.71 ± 69.49 kg and 107.86 ± 28.85 kg, respectively; however, this difference was not considered statistically significant (*p* = 0.36). All umbilical hernias were deemed not incarcerated after ultrasonographic evaluation. The median (Q1, Q3) hernia window diameters were 2.7 (2.2, 3.2) cm in the RSB group and 3.5 (3.5, 5.2) cm in the control group. The hernia window diameter was statistically longer in the control group than in the RSB group (median diff. 1.3 cm, 95% CI 0.30–3.00 cm, *p* = 0.004).

#### 3.2.1. Intraoperative parameters

Anesthesia and surgery times were similar in both groups. The cumulative dose of xylazine and ketamine used during general anesthesia did not differ between treatments. No physiologic parameters varied during surgery between groups. Indeed, the variations in HR and respiratory rate were minimal. We found a weak correlation (Pearson’s correlation coefficient 0.36) between the cumulative intraoperative dose of ketamine received and the HR in the calves. However, even when accounting for ketamine, HR did not differ significantly between groups (coef. 3.72, SE 3.77, 95% CI −4.59 to 12.02, *p* = 0.34). In both groups, 50% of the animals could maintain sternal position 5 min after the end of surgery. When controlling for the total amount of xylazine and ketamine given during the procedure, there was no statistically significant difference in time to achieve sternal position between groups (HR 0.47, SE 0.32, 95% CI 0.12 to 1.80, *p* = 0.28). The median (Q1, Q3) time required to stand was 5 (3, 9) min in calves receiving RSB and 8 (5, 20) min in calves receiving a sham injection. Even though the difference appeared clinically relevant, it was not statistically significant (HR 2.91, SE 2.00, 95% CI 0.76 to 11.15, *p* = 0.12). Intraoperative data are summarized in Table 1.

**Table 1:** Intraoperative parameters.

	RSB group	Control	95% CI	*p* value
Length anesthesia (min)	45 (45, 60)	40 (40, 53)	−17 to 5	0.19
Length surgery (min)	15 (15, 20)	15 (10, 20)	−9 to 5	0.43
Total xylazine (mg/kg)	0.04 (0.04, 0.04)	0.04 (0.03, 0.04)	−0.01 to 0.01	0.94
Total ketamine (mg/kg)	4.5 (3.50, 5.50)	4.5 (4.00, 6.00)	−0.62 to 1.94	0.28
Mean HR (bpm)	75.89 ± 9.01	74.03 ± 4.56	−10.17 to 6.46	0.64
Mean *f*_R_ (bpm)	37.65 ± 8.21	41.69 ± 9.46	−6.29 to 14.35	0.41
Time to sternal (min)	5.00 (2.00, 10.00)	5.00 (3.00, 5.00)	0.12 to 1.80	0.28
Time to stand (min)	5.00 (3.00, 9.00)	8.00 (5.00, 20.00)	0.76 to 11.15	0.12

CI, confidence interval; HR, heart rate; RSB, rectus sheath block; f_R_, respiratory rate.

Summary table of intraoperative variables in calves receiving the bilateral RSB with 0.3 ml/kg 0.25% bupivacaine (Bupivacaina Recordati; Recordati S.p.A., Italy) containing dexmedetomidine (1 μg/ml; Dexdomitor 0.5 mg/ml; Zoetis Inc., United States) and in controls. Data are presented as mean ± SD or median [interquartile range (Q1, Q3)] if normally or not normally distributed, respectively.

#### 3.2.2. Pain assessment

##### 3.2.2.1. Pain scores

**Figure 3:**
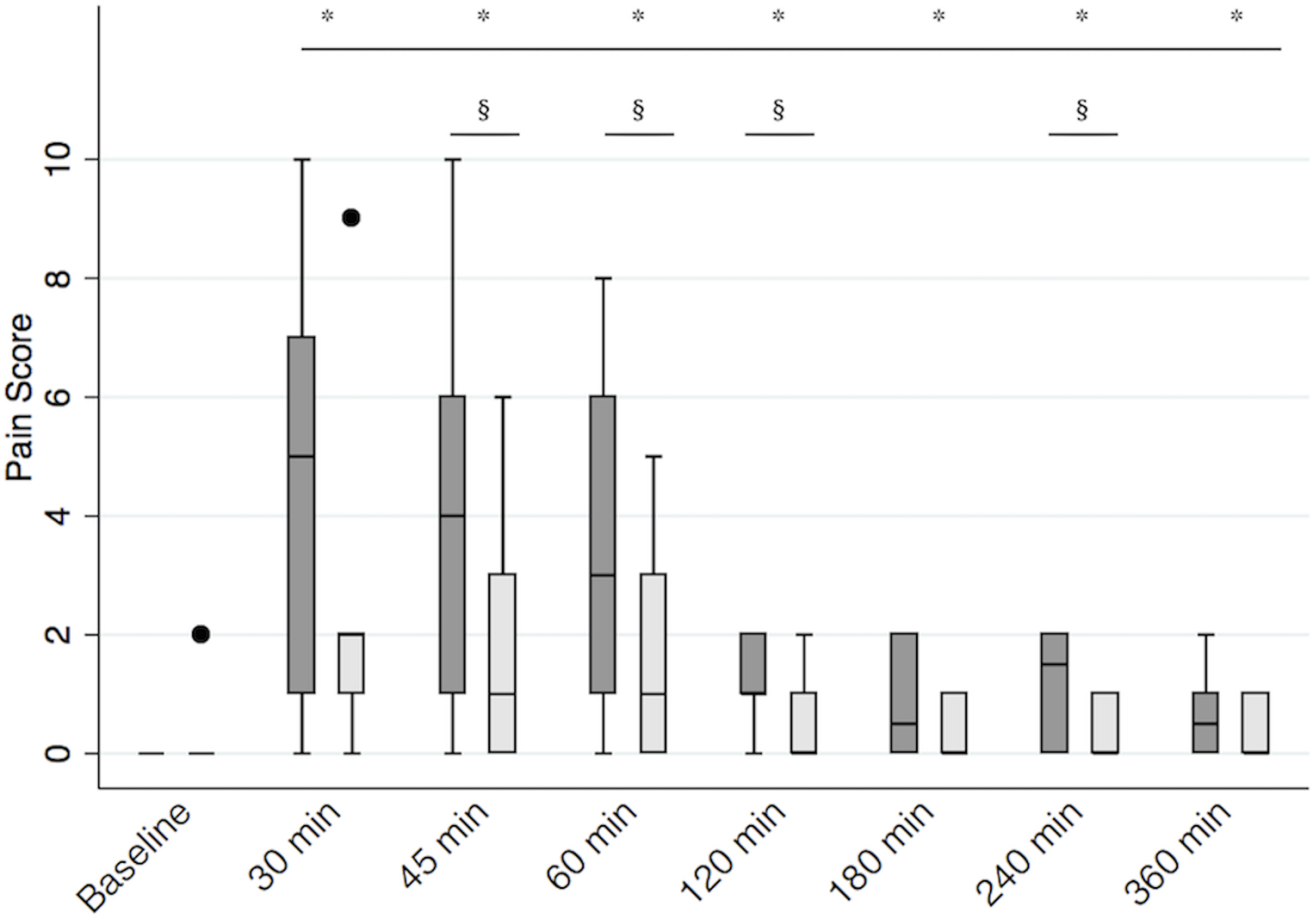
Postoperative pain scores. Boxplot representing median [interquartile range (Q1, Q3)] postoperative pain scores in calves receiving the bilateral RSB (light gray) with 0.3 ml/kg 0.25% bupivacaine (Bupivacaina Recordati; Recordati S.p.A., Italy) containing dexmedetomidine (1 μg/ml; Dexdomitor 0.5 mg/ml; Zoetis Inc., United States) and in controls (dark gray). *significantly different from baseline; § significantly different between groups. RSB, rectus sheath block.

The median (Q1, Q3) pain score at baseline was 0 (0, 0) in both groups (*p* = 0.32). When we looked at the pain scores in the two groups over time, we found that sedation had a significant impact on the pain score (*p* = 0.02), so it was included in the final regression model. The final multivariable regression model was significant (*p* < 0.01). When considering all variables in the model, pain scores were overall significantly higher than baseline for the entire study period (all *p*’s < 0.01). We found a significant interaction between treatment and time. Calves receiving RSB recorded lower pain scores between 45 and 120 min after recovery (*p* < 0.05) and at 240 min after recovery (*p* = 0.02) (Figure 3). The median (Q1, Q3) pain scores at 45, 60, 120, and 240 min were 1 (0, 3), 1 (0, 3), 0 (0, 1), and 0 (0, 1) in the RSB group and 4 (1, 6), 3 (1, 6), 1 (1, 2), and 1.5 (0, 2) in the control group, respectively.

##### 3.2.2.2. Mechanical threshold

**Figure 4:**
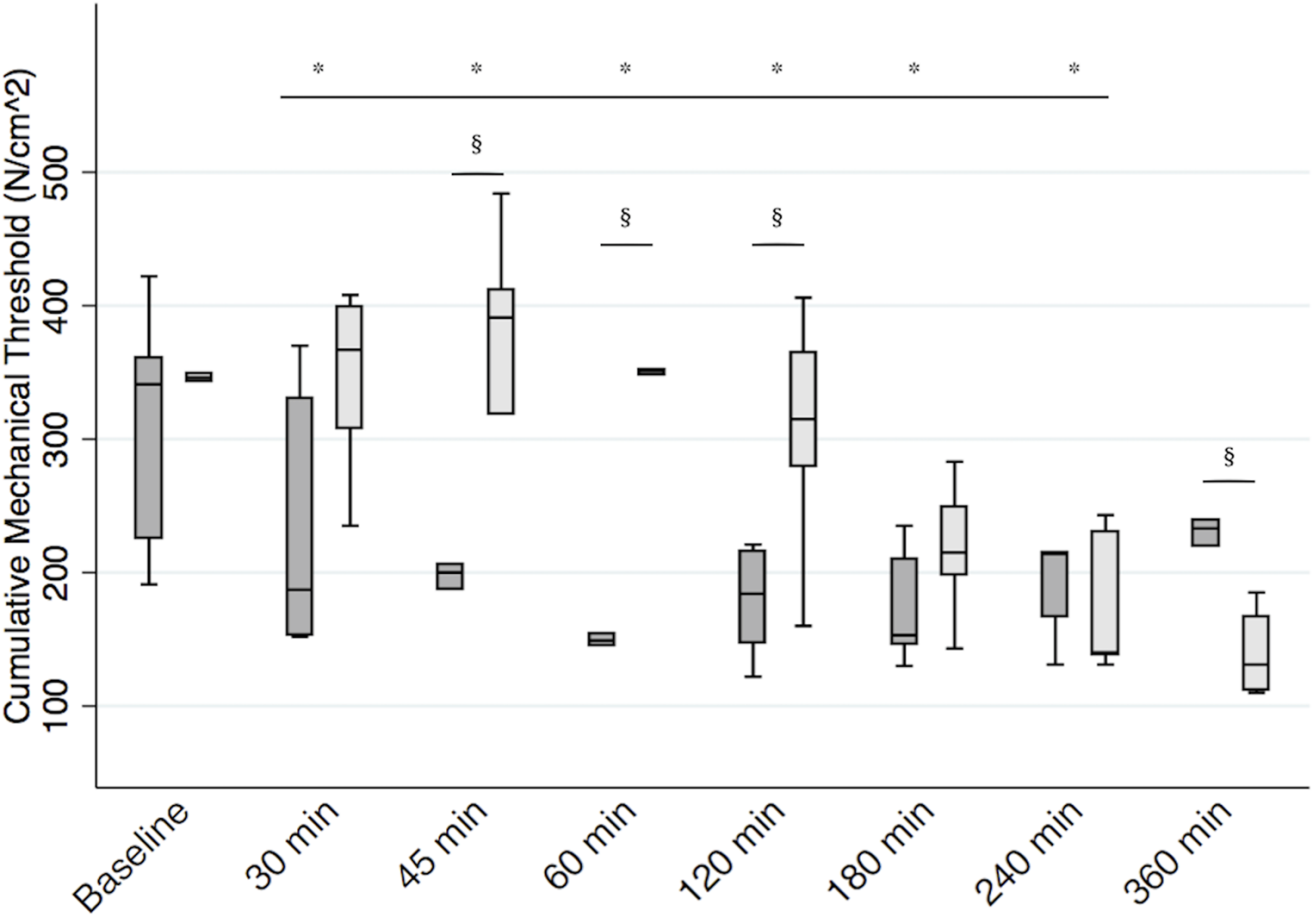
Postoperative mechanical threshold. Boxplot representing median [interquartile range (Q1, Q3)] cumulative mechanical threshold at the incision (expressed in N/cm^2^) in calves receiving the bilateral RSB (light gray) with 0.3 ml/kg 0.25% bupivacaine (Bupivacaina Recordati; Recordati S.p.A., Italy) containing dexmedetomidine (1 μg/ml; Dexdomitor 0.5 mg/ml; Zoetis Inc., United States) and in controls (dark gray). *significantly different from baseline. §significantly different between groups. RSB, rectus sheath block.

There was no difference in cumulative mechanical threshold between the two groups at baseline (median diff. 5 N/cm^2^, 95% CI −174 to 79, *p* = 0.46). Baseline median (Q1, Q3) mechanical thresholds on the left and right sides of the hernia were 160.5 (127, 176) N/cm^2^ and 182 (128, 202) N/cm^2^, respectively. There was no difference in baseline mechanical thresholds between the two sides (*p* = 0.24). When considering all variables in the model, the cumulative mechanical threshold significantly decreased with respect to baseline for 240 min after recovery (all *p*’s < 0.05). We found a significant interaction between treatment and time (*p* < 0.01). In particular, calves receiving the RSB recorded a higher cumulative mechanical threshold than the control group between 45 and 120 min after surgery (*p* < 0.05). Median (Q1, Q3) cumulative mechanical thresholds at 45, 60, and 120 min were 391 (319, 412) N/cm^2^, 351 (348, 352) N/cm^2^, and 315 (279, 365) N/cm^2^ for calves receiving RSB and 200 (187, 206) N/cm^2^, 149 (145, 154) N/cm^2^, and 184 (147, 216) N/cm^2^ for calves receiving the sham block, respectively. Mechanical threshold then progressively decreased, and it was significantly lower in the RSB group [131 (111.5, 167) N/cm^2^] than in the control group [233 (220, 239) N/cm^2^] 360 min after recovery (*p* < 0.01) (Figure 4). When we considered the mechanical threshold of the left and right sides separately, the results did not change.

## 4. Discussion

The present study describes an ultrasound-guided, transverse, in-plane rectus sheath injection with methylene blue in calf cadavers and its clinical efficacy in providing perioperative analgesia after herniorrhaphy under general anesthesia in field conditions. Our study confirmed that the plane between the rectus abdominis muscle and its internal sheath could be easily identified and targeted with high-frequency ultrasonography using an in-plane technique, as described by Ferreira et al. (25). Moreover, we found that bilateral RSB with 0.25% bupivacaine HCl, with the addition of 1 µg/ml dexmedetomidine HCl, improves postherniorrhaphy analgesia in calves receiving a postoperative dose of flunixin meglumine IV.

Historically, perioperative analgesia for calves undergoing umbilical herniorrhaphy under field conditions was provided using only NSAIDs. Constant intravenous infusion of morphine–lidocaine–ketamine proved effective in providing analgesia 24 h following umbilical herniorrhaphy in Holstein calves (17). However, its use is limited to hospital settings. Scarce peer-reviewed studies have described locoregional techniques for desensitizing the abdominal wall in large animal species, specifically ruminants. The caudal epidural injection of 2% xylazine at a dose of 0.2 mg/kg, diluted with 2% procaine solution to a final volume of 0.6 ml/kg, proved effective in providing visceral analgesia for umbilical herniorrhaphy in 10 not-sedated German Holstein calves (9). However, sacrococcygeal epidural anesthesia has potential complications such as inconsistent efficacy, hypotension, infection, inadvertent subarachnoid injection, and bilateral pelvic limb paralysis. The ventromedial branches of T10–T12 spinal nerves innervate the abdominal wall around the umbilicus in cattle (18, 19). Therefore, the peripheral blockade of these nerves may provide perioperative analgesia for calves undergoing omphalectomy. Peripheral blocks can be easily performed with the animal under sedation or general anesthesia and in dorsal recumbency as surgical preparation occurs. An ultrasound-guided transversus abdominis plane (TAP) block was described in calf cadavers with both a lateral and subcostal approach and one-point injection of either 0.2 or 0.4 ml/kg 1% toluidine blue solution (34). While the visualization of the injection plane and feasibility were excellent for most injections, both approaches failed to stain the ventral branch of T10, making the technique useless for cranial abdominal analgesia. This is corroborated by the fact that the TAP block performed on two calves undergoing umbilical surgery failed to decrease inhalant or rescue analgesic requirements (35). Similarly to horses, a three-point injection technique may be required to produce consistent anesthesia of the ventral abdominal wall of calves (36). However, multiple injections may add to anesthesia time and decrease clinical efficiency, especially in field conditions.

Injection within the rectus sheath has recently been described in 16 calf cadavers using either 0.25 or 0.5 ml/kg 1% methylene blue per hemiabdomen (25). The technique used was similar to the one described in the present work. However, the investigators reported more consistent complete staining and a significantly greater number of completely stained ventral branches of spinal nerves using the larger volume of injectate than the smaller volume. Specifically, nerves T10–T12 were completely stained in 62.5%, 56.3%, and 37.5% of 0.25 ml/kg methylene blue injections. Our study could not isolate the thoracic nerves’ ventral branches. However, the craniocaudal and lateral spreads within the rectus sheath were similar to what was described by Ferreira et al. (25). The volume for the injection used in the current study was optimized based on cadaveric descriptions of a canine RSB and volumes used for RSB in human patients (21, 24, 26). When the study was performed, the technique was not described in calves yet (25). Moreover, a TAP block performed with 0.5 ml/kg volume in ponies provided concern for inadvertent pelvic limb weakness (37).

Despite these incongruences, 0.3 ml/kg 0.25% bupivacaine HCl per hemiabdomen provided superior perioperative analgesia than the sole use of NSAIDs when measured with a validated composite pain scale and quantitative sensory testing (algometry) at specific postoperative time points. Our positive results could be explained by the anesthetic solution spread around the incision. Local infiltration of the umbilicus in calves undergoing herniorrhaphy under injectable anesthesia provided sufficient anesthesia/analgesia for surgery (9). Nevertheless, similar to the latter study, repeated doses of ketamine and xylazine were necessary to maintain a constant level of anesthesia in the current study. Heart and respiratory rates during anesthesia were similar to those reported by Offinger et al. (9). While we did not obtain arterial blood samples, SpO_2_ measures were never recorded below 95%. The other hypothesis that may explain why a smaller volume per unit of body weight was sufficient to provide perioperative analgesia is that, in a given species, the increase in body weight with aging is associated with a reduction of surface area per unit of body weight (38). In other words, the body surface area of our calves, weighing on average over 130 kg, was proportionally smaller than that of calves used by Ferreira et al. (25), potentially allowing for more ventral branches of spinal nerves stained.

The first limitation of the study is the lack of complete intraoperative monitoring due to the field conditions. However, this recreates a realistic scenario of the conditions general practitioners commonly perform these surgeries. On the other hand, the equipment and skills needed to perform ultrasound-guided fascial local blocks may not be available to the general practitioner. Given the anatomy, local anesthetic injections within the T10–T12 intercostal spaces, similar to the Caudal Intercostal Block for Abdominal Surgery (CIBAS) described in horses (39), may allow for a more accessible technique to provide perioperative analgesia in calves undergoing umbilical herniorrhaphy under field conditions and should be further explored. Finally, the study did not have a positive control group, with calves receiving an injection of local anesthetics on the incision line. While human literature supports the superiority of the RSB vs. line block for pediatric herniorrhaphy surgery (23), our study was meant to demonstrate the efficacy of the newly described RSB and not its superiority vs. a line block. Similarly, the study did not compare different volumes of local anesthetic agents, so it did not aim to find an optimum volume for an umbilical blockade. Both these latter limitations leave the scenario open to further investigation.

## 5. Conclusion

In conclusion, this study described the effective perioperative analgesic effects of bilateral ultrasound-guided RSB with 0.3 ml/kg 0.25%bupivacaine HCl, and the adjunct of 1 µg/ml dexmedetomidine HCl, in calves undergoing herniorrhaphy under injectable general anesthesia in field conditions. However, further studies are warranted to better assess the clinical usefulness of this technique and determine an optimal volume of local anesthetic agents in live animals.

## Data Availability

The raw data supporting the conclusions of this article will be made available by the authors, without undue reservation.
