# Supplementary material for: Clinical efficacy of an ultrasound-guided bilateral rectus sheath block for umbilical hernia repair in calves: A prospective randomized trial
Source: Front Pain Res (Lausanne). 2023 Feb 13;4:1051504. doi: 10.3389/fpain.2023.1051504 (PMC9969087; doi:10.3389/fpain.2023.1051504)
Supplement: Supplementary file 1 [file Table1.docx]

Supplementary Material

# Supplementary Data

| **Parameters** | **Score/Criterion** |
| --- | --- |
| **Locomotion** | (0) Walking with no obviously abnormal gait  (1) Walking with restriction, ma be with hunched back and/or short steps  (2) Reluctant to stand up, standing up with difficulty or not walking |
| **Interactive behaviour** | (0) Active: attention to tactile and/or visual and/or audible environmental stimuli; when near other animals, can interact with and/or accompany the group  (1) Apathetic: may remain close to other animals, but interacts little when stimulated  (2) Move less frequently in the pasture or only when stimulated |
| **Activity** | (0) Moves normally  (1) Restless, moves more than normal or lies down and stands up with frequency  (2) Move less frequently in the pasture or only when stimulated |
| **Appetite** | (0) Normorexia and/or rumination  (1) Hyporexia  (2) Anorexia |
| **Miscellaneous behaviours** | - Wagging the tail abruptly and repeatedly - Licking the surgical wound - Moves and arches the back when in standing posture - Kicking/foot stamping - Hind limbs extended caudally when in standing posture - Head below the line of spinal column - Lying down in ventral recumbency with full or partial extension of one or both hind limbs. - Lying down with the head on/close to the ground - Extends the neck and body forward when lying in ventral recumbency   (0) All of above-described behaviours are absent  (1) Presence of 1 of the behaviours described above  (2) Presence of 2 or more of the behaviours described above |

**Supplementary Table 1.** UNESP-Botucatu unidimensional pain scale used at different time intervals in calves receiving or not ultrasound-guided rectus sheath block.
